# Evaluation of primary school teachers’ use and perceptions of artificial intelligence in primary school mathematics instruction: a mixed-methods study

**DOI:** 10.3389/fpsyg.2026.1741116

**Published:** 2026-09-18

**Authors:** Fatih Mehmet Ciğerci, Neşe Uygun

**Affiliations:** 1 Department of Primary Education, Faculty of Education, Harran University, Şanlıurfa, Türkiye; 2 Department of Primary Education, Faculty of Nizip Education, Gaziantep University, Gaziantep, Türkiye

**Keywords:** artificial intelligence, educational technology, mathematics teaching, primary school, teacher perceptions

## Abstract

**Introduction:**

This study examined primary school teachers’ reported perceptions and self-reported uses of artificial intelligence (AI) in primary school mathematics instruction, with particular attention to how perceptions vary according to teachers’ demographic characteristics and AI-related experiences.

**Methods:**

The study employed an explanatory sequential mixed-methods design and was conducted with 302 primary school teachers in Türkiye during the 2024–2025 academic year. Quantitative data were collected using the Scale for Assessing Teachers’ Perceptions of Artificial Intelligence in Education. The quantitative findings were subsequently elaborated through semi-structured interviews with teachers who had prior AI-related training.

**Results:**

Teachers reported moderately positive overall perceptions of AI in mathematics instruction. Perception scores varied according to gender, professional experience, educational background, frequency of AI use, and prior AI training, although these differences were not consistent across all dimensions and some were practically modest. More frequent AI use and prior AI-related training were associated with more positive perceptions. Qualitative findings showed that teachers primarily positioned AI as a supportive resource for lesson planning, content and material development, visualization, problem solving, formative assessment, feedback, and out-of-class learning. ChatGPT, GeoGebra, and Photomath were among the most frequently mentioned tools. Teachers also identified concerns regarding age appropriateness, technological infrastructure, data privacy, reliability, teacher competence, and students’ possible over-reliance on ready-made responses.

**Discussion:**

The findings indicate a generally favorable but cautious orientation toward AI in primary mathematics education. Teachers’ willingness to use AI and positive attitudes do not necessarily reflect extensive or pedagogically established classroom integration. Rather than demonstrating instructional effectiveness, the study provides a context-specific account of the perceived pedagogical opportunities, practical constraints, and conditions associated with responsible AI integration in primary mathematics instruction.

## 1 Introduction

Primary school mathematics instruction requires teachers to respond to substantial differences in students’ prior knowledge, learning pace, conceptual understanding, and problem-solving strategies. Artificial intelligence (AI) has attracted growing attention in this context because AI-supported systems can potentially provide differentiated practice, generate multiple representations of mathematical ideas, identify patterns in student responses, and offer timely feedback. The recent expansion of generative artificial intelligence (GenAI) has further broadened these possibilities by enabling teachers to generate instructional materials, adapt tasks, develop explanations, and interact with digital tools in increasingly flexible ways. At the same time, AI in education encompasses a heterogeneous range of technologies, from intelligent tutoring and learning analytics systems to generative applications such as ChatGPT. Their educational value therefore depends not simply on technical capability, but on the ways in which particular tools are selected and pedagogically mediated within specific instructional contexts (Hwang, 2022; Drijvers and Sinclair, 2024; Alfarwan, 2025).

Teachers are central to this process. Whether AI becomes a meaningful instructional resource depends partly on teachers’ willingness to engage with these technologies, their prior experience, their ability to evaluate AI-generated outputs, and their capacity to align technology use with curricular and developmental goals. Recent K–12 research suggests that teachers often recognize the potential of AI for lesson planning, personalization, resource development, and instructional efficiency, while simultaneously expressing uncertainty about reliability, ethics, curricular alignment, and their own preparedness (Cheah and Kim, 2026; Cheah et al., 2025; Filiz et al., 2025). Familiarity with AI also appears to be associated with more favorable perceptions. For example, Cheah and Kim (2026) found considerable variation in K–12 STEM teachers’ familiarity with GenAI and reported a positive relationship between familiarity and perceptions of its educational use. Similarly, recent research in Türkiye indicates that teachers’ engagement with AI is shaped not only by perceived usefulness but also by technical constraints, pedagogical considerations, ethical concerns, and access to professional support (Filiz et al., 2025). These findings suggest that positive attitudes toward AI should not be equated with established or pedagogically sophisticated classroom integration.

This distinction is particularly important in primary mathematics education. Although research on AI and GenAI in K–12 education is growing rapidly, the evidence base remains uneven across educational levels, subject areas, and participant groups. A recent systematic review of empirical GenAI research in K–12 settings found that existing studies have been concentrated disproportionately at the secondary level and among preservice teachers, leaving comparatively less evidence on primary-school contexts and practicing teachers (Alfarwan, 2025). Mathematics-specific research has likewise expanded, but much of it has focused on student performance, intelligent tutoring, particular mathematical tasks, or relatively bounded applications of GenAI. Emerging studies with mathematics teachers illustrate the potential of AI to support task design, professional noticing, and pedagogical reasoning (Galiç et al., 2025; Segal and Klemer, 2025), while recent reviews of ChatGPT in school mathematics emphasize both its potential for explanation and problem solving and the risks associated with inaccurate output, overreliance, and insufficient pedagogical mediation (Turmuzi et al., 2026). What remains less clear is how practicing primary school teachers perceive AI specifically within mathematics instruction, how these perceptions vary according to their backgrounds and AI-related experiences, and how they position AI across different stages of everyday instructional practice.

The Turkish context provides a particularly relevant setting for investigating these questions. Türkiye has recently formalized its policy orientation toward AI through the Ministry of National Education’s Artificial Intelligence in Education Policy Document and Action Plan (2025–2029), which emphasizes teacher competence, responsible use, educational applications, and institutional capacity (Ministry of National Education, 2025). Recent research with Turkish K–12 teachers has begun to identify the factors that facilitate or constrain AI integration across subject areas (Filiz et al., 2025). However, subject-specific evidence concerning primary mathematics remains limited. This matters because the pedagogical value and risks of AI may differ according to disciplinary demands. In mathematics, for example, rapid answer generation can support feedback and demonstration but may also reduce opportunities for reasoning, persistence, representation, and independent problem solving when it is not carefully mediated.

Against this background, the study focuses specifically on primary school teachers’ reported perceptions and self-reported uses of AI in mathematics instruction. It makes three related contributions. First, it examines AI within a subject- and level-specific context rather than treating educational AI use as a generic phenomenon. Second, it considers teachers’ perceptions across multiple dimensions—willingness to use AI, attitudes toward AI, professional expectations, and personal experiences—and investigates their variation according to gender, professional experience, educational background, frequency of AI use, and prior AI training. Third, through an explanatory sequential mixed-methods design, quantitative patterns are interpreted alongside teachers’ qualitative accounts of the roles, opportunities, and constraints they associate with AI across instructional practice. Importantly, the study does not evaluate the effectiveness of AI for improving mathematics achievement or observed teaching quality. Its contribution concerns how teachers perceive, describe, and report their engagement with AI in primary mathematics classrooms.

Accordingly, the study addresses the following research questions:

1. What are primary school teachers’ reported perceptions of AI in primary school mathematics instruction across the dimensions of willingness to use AI, attitudes toward AI, professional expectations, and personal experiences?
2. Do these reported perceptions differ significantly according to gender, professional experience, educational background, frequency of AI use, and prior AI training?
3. How do primary school teachers describe the pedagogical roles of AI across lesson planning, lesson implementation, problem solving, assessment, feedback, and out-of-class learning in primary mathematics instruction?
4. What opportunities and constraints do teachers report regarding the responsible integration of AI into primary school mathematics teaching?

## 2 Literature review

### 2.1 The role of artificial intelligence in education

Artificial intelligence in education encompasses technologies with different levels of automation, interaction, and pedagogical functionality, including intelligent tutoring systems, adaptive learning environments, learning analytics, automated assessment, and, more recently, GenAI applications. Across these forms, AI has been associated with possibilities for individualized support, rapid feedback, instructional resource development, and data-informed decision-making. However, recent K–12 literature suggests that educational AI should not be understood as a uniform intervention whose effects can be separated from its context of use. A systematic review of GenAI research in K–12 education found considerable diversity in applications and study designs and showed that the empirical knowledge base is still developing, particularly outside secondary and preservice-teacher contexts (Alfarwan, 2025). This emerging evidence therefore supports a shift from asking whether AI is inherently beneficial to examining what functions particular technologies perform, for whom, under what conditions, and with what form of teacher mediation.

One of the most consistent themes in recent research concerns teacher preparedness. K–12 teachers generally report recognizing potential benefits of AI but differ substantially in their familiarity with the technologies and confidence in incorporating them into practice. Cheah and Kim (2026), for example, found varied levels of GenAI familiarity among STEM teachers and identified professional development as a central support need. A broader investigation of K–12 teachers similarly showed that preparedness and actual practices were accompanied by persistent barriers to classroom integration (Cheah et al., 2025). In Türkiye, Filiz et al. (2025) found that teachers valued AI for its efficiency, adaptability, interactivity, personalization, and support for lesson planning, while identifying technical difficulties, curricular misalignment, ethical concerns, and contextual barriers. Together, these studies indicate that acceptance of AI and capacity to use it pedagogically are related but distinct dimensions of teacher readiness.

Recent scholarship has therefore placed increasing emphasis on AI literacy rather than basic technical familiarity alone. AI literacy involves understanding the capabilities and limitations of AI, using AI-supported tools purposefully, critically evaluating their outputs, and recognizing ethical implications related to issues such as bias, privacy, transparency, and accountability. Research with teachers indicates that acceptance-related variables are closely associated with AI literacy. Deshen et al. (2026), for example, found positive associations between teachers’ AI acceptance and AI literacy, with willingness to use AI among the strongest explanatory variables. Their results also indicated differences associated with educational background and professional use of AI. Such findings are particularly relevant to educational settings because teachers must make judgments not only about whether a technology is convenient to use, but also about whether its outputs are pedagogically sound, developmentally appropriate, and compatible with instructional objectives.

These issues become more consequential in compulsory schooling, where students’ developmental characteristics and institutional responsibilities place additional constraints on technology use. International guidance has consistently highlighted data privacy, algorithmic bias, transparency, unequal access, and the reliability of AI-generated information as central concerns in educational implementation (UNESCO, 2021a,b, 2023). Recent K–12 research likewise documents concerns related to technical access, ethical uncertainty, curriculum compatibility, and teacher competence (Cheah et al., 2025; Filiz et al., 2025). Accordingly, the educational value of AI cannot be separated from questions of infrastructure, professional knowledge, governance, and equity. These concerns are especially salient for younger students, who may have less capacity to independently assess the reliability or appropriateness of AI-generated responses.

For these reasons, current perspectives increasingly frame AI as a resource that may augment rather than replace teachers’ professional work. AI can potentially reduce the time required for routine instructional tasks, support differentiated resource development, and provide additional information about student learning, but teachers remain responsible for interpreting these outputs and determining how they should be used in relation to learners, curricula, and classroom contexts. This human-centered position also highlights an important distinction between technological availability and pedagogical integration: access to AI does not in itself indicate that the technology has become a meaningful part of instruction. Teacher judgment, subject knowledge, pedagogical knowledge, and opportunities for professional learning continue to mediate this process.

### 2.2 Artificial intelligence in mathematics education

Mathematics presents a particularly relevant context for AI-supported instruction because learning frequently involves cumulative conceptual development, multiple forms of representation, repeated practice, error diagnosis, and individualized feedback. Earlier AI research in mathematics has demonstrated the potential of intelligent tutoring and adaptive learning systems to respond to individual performance and provide targeted guidance. Meta-analytic evidence at the elementary level has also suggested positive effects of AI-supported mathematics instruction on achievement, although these effects depend on the nature of the technology and the conditions under which it is implemented (Hwang, 2022). The growing availability of GenAI adds new possibilities because teachers and learners can now interact with systems capable of generating explanations, examples, problems, representations, and feedback in natural language.

Recent mathematics-education research, however, also shows why pedagogical mediation is essential. GenAI can generate step-by-step responses and alternative explanations, but mathematically plausible language does not guarantee conceptual accuracy or pedagogical appropriateness. A recent systematic review of ChatGPT in school mathematics identified opportunities related to problem solving, explanation, personalization, and instructional support alongside concerns about erroneous responses, dependence on generated solutions, reduced cognitive effort, and the need for teachers to critically evaluate outputs (Turmuzi et al., 2026). This tension is particularly relevant at the primary level, where the purpose of mathematics instruction is not simply to obtain correct answers but to develop number sense, reasoning, conceptual relationships, representations, and problem-solving strategies.

The teacher-facing functions of AI are therefore as important as student-facing applications. AI can potentially support planning by generating examples, adapting tasks to different readiness levels, suggesting alternative representations, and assisting in the construction of assessment materials. Recent mathematics-specific studies provide evidence of this expanding role. Segal and Klemer (2025) examined mathematics teachers’ interactions with GenAI while planning primary-school geometry tasks in technology-rich environments, demonstrating the relevance of pedagogical, technological, and mathematical knowledge when teachers work with generative systems. Galiç et al. (2025), through scenario-based work with in-service mathematics teachers, likewise showed how interaction with an AI chatbot can become part of teachers’ professional noticing and pedagogical reasoning. These studies are important because they shift attention from AI as an autonomous instructional agent toward the ways teachers interpret, question, adapt, and contextualize AI-generated material.

AI may also support formative assessment and instructional decision-making by providing personalized support and rapid feedback and by generating information that can inform teachers’ decisions about student learning (Toptaş, 2024). Learning analytics and teacher dashboards have already illustrated how digital information about student progress can inform instructional decisions in mathematics (Moed-Abu Raya and Olsher, 2024). GenAI may extend these possibilities through more flexible content and feedback generation. Nevertheless, increased efficiency does not automatically imply improved instructional quality. Teachers must determine whether generated explanations reflect appropriate mathematical reasoning, whether feedback supports rather than bypasses productive struggle, and whether tasks remain aligned with curriculum expectations and students’ developmental levels.

The emerging literature consequently suggests a dual character of AI in mathematics education. On the one hand, AI may expand teachers’ capacity to differentiate instruction, visualize abstract ideas, generate materials, provide feedback, and support problem solving. On the other hand, poorly mediated use may encourage answer-seeking rather than reasoning, obscure errors behind fluent explanations, increase dependence on external support, or reproduce existing inequities in access to digital resources. These tensions become especially significant in primary education because younger learners require greater instructional scaffolding and may be less equipped to independently evaluate AI outputs. The role of the teacher therefore remains central not only to the effectiveness of AI-supported mathematics instruction but also to its developmental and ethical appropriateness.

Despite rapidly growing interest, there remains comparatively little evidence that combines these issues from the perspective of practicing primary school teachers themselves. Recent K–12 and mathematics-specific studies have substantially advanced understanding of teacher readiness, AI familiarity, GenAI-supported task design, and the opportunities and challenges of AI use (Cheah and Kim, 2026; Filiz et al., 2025; Galiç et al., 2025; Segal and Klemer, 2025). Yet fewer studies have simultaneously examined multidimensional teacher perceptions, differences associated with AI experience and background variables, and teachers’ accounts of where and how AI is positioned across the full instructional process in primary mathematics. The present study addresses this intersection.

### 2.3 Theoretical and conceptual framework

The study is informed by two complementary perspectives: the Technology Acceptance Model (TAM) and technological-pedagogical knowledge frameworks represented by TPACK and its AI-related extensions. These frameworks are used as interpretive lenses rather than as models that are fully operationalized or statistically tested in the present study. TAM proposes that users’ beliefs about a technology—particularly its perceived usefulness and ease of use—shape attitudes and behavioral intentions related to adoption (Davis, 1989). This perspective is relevant to the present study because teachers’ willingness to use AI, attitudes toward AI, frequency of use, prior training, and personal experiences represent different aspects of engagement with an emerging technology. Recent teacher research provides additional support for considering acceptance and experience together. Cheah and Kim (2026) identified an association between teachers’ familiarity with GenAI and their perceptions of its educational integration, while Deshen et al. (2026) found that willingness and other acceptance-related variables were strongly associated with teachers’ AI literacy. These findings suggest that teachers’ perceptions may develop in interaction with experience and exposure rather than functioning as stable attitudes independent of practice.

Technology acceptance alone, however, cannot explain whether AI is used in pedagogically meaningful ways. TPACK emphasizes that effective technology integration depends on the interaction of technological, pedagogical, and content knowledge rather than technological knowledge in isolation (Mishra and Koehler, 2006). Recent work on AI in K–12 education has extended this argument by examining teachers’ readiness for AI through TPACK-related perspectives. Yue et al. (2024), in a study involving K–12 teachers, identified substantial gaps in AI-related technological and content knowledge and demonstrated relationships between teachers’ knowledge profiles and attitudes toward AI education. Emerging AI-TPACK perspectives similarly emphasize that teachers must be able to evaluate the educational affordances and limitations of AI, connect AI-supported practices to subject-specific learning goals, and make informed decisions regarding ethical and responsible use. Recent work on AI-focused professional development reinforces the need to treat this knowledge as pedagogical and ethical as well as technical (Mah et al., 2026).

Taken together, these perspectives provide a useful conceptual basis for interpreting the mixed-methods findings of the present study. TAM helps explain variation in teachers’ willingness, attitudes, and engagement with AI, whereas TPACK and AI-related competency perspectives help interpret how teachers describe translating that engagement into mathematics-specific instructional practices. The distinction is particularly important because a teacher may perceive AI positively without possessing the experience or pedagogical knowledge required for sustained classroom integration. Conversely, greater familiarity and training may expand teachers’ capacity to judge when AI is useful, when its outputs require modification, and when its use may be inappropriate. In the present study, these frameworks therefore connect the quantitative examination of perceptions and background variables with the qualitative investigation of lesson planning, implementation, problem solving, assessment, feedback, and reported opportunities and constraints. They are not used to infer unmeasured constructs such as AI literacy or technological competence; rather, they provide a theoretically grounded vocabulary for interpreting the relationships and instructional accounts documented in the study.

## 3 Materials and methods

The primary aim of this study was to examine primary school teachers’ reported perceptions of artificial intelligence (AI) in primary school mathematics instruction and their self-reported views regarding how AI is used in instructional practice. Within this scope, teachers’ perception scores were analyzed in relation to gender, professional experience, educational background, frequency of AI use, and prior AI training, and these quantitative patterns were further interpreted through teachers’ qualitative accounts. The study does not evaluate the direct effect of AI on student achievement or instructional effectiveness; rather, it focuses on teachers’ reported perceptions, reported uses, and pedagogical interpretations of AI in the context of primary mathematics instruction.

Considering this purpose, a mixed-methods research design was employed. Mixed-methods research enables researchers to combine quantitative and qualitative evidence in order to generate a broader and more contextually grounded understanding of a research problem (Creswell, 2014). Accordingly, this study adopted an explanatory sequential design, in which quantitative data are collected and analyzed first, followed by qualitative data that help explain, elaborate, and contextualize the quantitative findings (Creswell and Guetterman, 2024). The selection of this design was based on the study’s aims of identifying the overall distribution of teachers’ AI-related perception scores in primary mathematics instruction and their variation according to selected variables, and explaining these patterns through in-depth teacher accounts of practice, opportunity, and constraint.

In this study, the quantitative phase employed a descriptive survey design to identify teachers’ reported perceptions of AI in primary mathematics instruction. The qualitative phase used a phenomenological approach to explore how teachers who had prior AI training described their experiences and interpretations of AI-supported mathematics teaching.

### 3.1 Study group

The study group was determined in line with the explanatory sequential mixed-methods design of the research. In the quantitative phase, the study included 302 in-service primary school teachers working in Türkiye during the 2024–2025 academic year. Participants were recruited through a convenience sampling approach based on voluntary online participation. The survey link was distributed via professional teacher networks and institutional communication channels. To be included in the quantitative phase, participants had to be actively employed as a primary school teacher teaching Grades 1–4 during the data collection period and provide informed consent to participate in the study. Responses from teachers who were not actively teaching during data collection or who submitted incomplete forms were excluded from the analysis. The socio- demographic characteristics of the quantitative participants are presented in Table 1.

**TABLE 1:** Socio-demographic characteristics of the quantitative sample (*N* = 302).

Variable	Category	N	%
Gender	Male	90	29.8%
	Female	212	70.2%
	Total	302	100.0%
Professional experience	1–5 years	69	22.8%
	6–10 years	33	10.9%
	11–15 years	57	18.9%
	16–20 years	65	21.5%
	21 years and more	78	25.8%
	Total	302	100.0%
Educational background	Bachelor’s degree	190	62.9%
	Master’s degree	98	32.5%
	Doctorate	14	4.6%
	Total	302	100.0%
Level of AI use	Never use	54	17.9%
	Rarely use	75	24.8%
	Sometimes use	108	35.8%
	Frequently use	65	21.5%
	Total	302	100.0%
Status of receiving training on AI	Received training	58	19.2%
	Did not receive training	244	80.8%
	Total	302	100.0%

Percentages may not total 100 due to rounding.

As shown in Table 1, 70.2% of the participants were female (*n* = 212) and 29.8% were male (*n* = 90). In terms of professional experience, the largest group consisted of teachers with 21 or more years of experience (25.8%, *n* = 78), followed by those with 16–20 years (21.5%, *n* = 65) and 1–5 years (22.8%, *n* = 69). Regarding educational background, 62.9% of the participants held a bachelor’s degree (*n* = 190), 32.5% held a master’s degree (*n* = 98), and 4.6% held a doctoral degree (*n* = 14). With respect to AI use, 35.8% reported that they sometimes used AI (*n* = 108), 21.5% frequently used it (*n* = 65), 24.8% rarely used it (*n* = 75), and 17.9% reported never using it (*n* = 54). In addition, 19.2% of the participants (*n* = 58) stated that they had received prior training related to AI, whereas 80.8% (*n* = 244) had not.

Although participants represented different professional-experience levels, educational backgrounds, and AI-use profiles, the sample was not designed to be statistically representative of all primary school teachers in Türkiye. Because participation was voluntary and online, the possibility of self-selection bias should be acknowledged. In particular, teachers who were more interested in digital technologies or AI may have been more likely to participate.

In the qualitative phase, semi-structured interviews were conducted with 10 volunteer teachers selected through purposive sampling from among the 58 teachers who had previously received AI-related training. This criterion-based purposive sampling strategy was used to obtain information-rich cases from participants expected to have greater familiarity with AI-supported instructional practices and to be better positioned to clarify the quantitative findings. The qualitative strand was therefore designed to provide explanatory depth rather than statistical generalization. Demographic characteristics of the qualitative participants are presented in Table 2.

**TABLE 2:** Demographic characteristics of the qualitative interview participants (*n* = 10).

Participant	Gender	Educational background	Professional experience	AI tools used
P1	Male	Master’s degree	4	GeoGebra, ChatGPT, Mathematicshan, Photomath
P2	Male	Doctorate	17	Mathigon
P3	Female	Bachelor’s degree	8	ChatGPT
P4	Male	Bachelor’s degree	4	ChatGPT, Photomath, Mathigon
P5	Female	Doctorate	17	Bing, Gemini, ChatGPT, Leonardo AI, ElevenLabs
P6	Male	Doctorate	15	ChatGPT, GeoGebra
P7	Female	Master’s degree	5	ChatGPT
P8	Male	Bachelor’s degree	3	ChatGPT, Matific, Sora
P9	Male	Master’s degree	13	Gemini, ChatGPT, Copilot
P10	Female	Master’s degree	12	ChatGPT, DreamBox Learning, DragonBox, Khan Academy, Buzzmath

All interview participants had previously received AI-related training and were selected through purposive sampling.

As shown in Table 2, most of the interview participants held postgraduate degrees, and all had received training in AI-related applications relevant to mathematics teaching, including AI and AI-supported tools such as ChatGPT, Mathigon, GeoGebra, and Mathematicshan.

### 3.2 Data collection tools and process

For the quantitative phase of the study, the *Scale for Assessing Teachers’ Perceptions of Artificial Intelligence in Education* was used. The scale was originally developed by Galindo-Domínguez et al. (2025) and later adapted into Turkish by Ciğerci and Uygun (2025). The Turkish adaptation study provided evidence of the instrument’s validity and reliability for use with Turkish teachers. The scale includes 25 items organized under four dimensions: willingness to use AI, attitudes toward AI, professional expectations related to AI, and personal experiences with AI. All items are rated on a 5-point Likert-type scale, with higher scores indicating more positive perceptions of AI in education.

The use of this instrument in the present study was considered appropriate because it captures multiple dimensions of teachers’ reported perceptions rather than focusing on a single attitudinal construct. Since the present study analyzes composite subscale and total scores rather than individual Likert items in isolation, the instrument provides a structured basis for examining multidimensional perception patterns. In the current sample, the internal consistency of the scale was re-examined using Cronbach’s alpha.

For the qualitative phase, data were collected through semi-structured interviews based on an interview form developed by the researchers. The interview protocol was reviewed by three experts, including one primary school teacher and two faculty members, and finalized accordingly. The final form consisted of seven core questions, with follow-up questions used when necessary to obtain greater depth and clarification. The interviews were conducted via Zoom, audio-recorded with participants’ consent, transcribed verbatim, and shared with participants for confirmation before analysis began.

### 3.3 Data analysis

#### 3.3.1 Quantitative data analysis

Quantitative data were analyzed using IBM SPSS Statistics 30.0. The analysis proceeded in five stages: data screening, descriptive analysis, assumption testing and reliability analysis, inferential statistics, and effect-size interpretation. First, the dataset was screened for completeness and suitability for analysis, and incomplete responses were excluded prior to statistical testing. Frequency and percentage values were calculated to describe participants’ demographic characteristics. In addition, arithmetic mean (M), standard deviation (SD), minimum (Min.), and maximum (Max.) values were computed to summarize the distribution of total-scale and sub-dimension scores.

Before conducting inferential analyses, distributional characteristics were examined. Because the analyses were performed on total and subscale composite scores rather than on single Likert items, and because the sample size was relatively large (*N* = 302), parametric tests were considered acceptable where assumptions were reasonably satisfied. Normality was initially evaluated through skewness and kurtosis coefficients for the total scale and each sub-dimension, and homogeneity of variance was assessed using Levene’s test before group comparisons were conducted. However, given the ordinal-response structure of Likert-type data, the interpretation of parametric results should be made cautiously.

The internal consistency of the scale was examined using Cronbach’s alpha (α). In the present study, the Cronbach’s alpha coefficient for the total scale was 0.954, indicating very high internal consistency. Although this value supports score reliability, coefficients at this level may also signal item overlap or redundancy.

To examine group differences, independent-samples *t*-tests were used for dichotomous variables such as gender and prior AI training, and one-way ANOVA was used for variables with three or more categories such as professional experience, educational background, and frequency of AI use. When significant differences were identified, Tukey’s HSD *post-hoc* test was used to determine the source of between-group differences. The level of statistical significance was set at *p* < 0.05. In addition to significance testing, effect-size indices were used to support interpretation. Cohen’s *d* was used for *t*-tests and eta squared (η^2^) was used for ANOVA results. In interpreting these results, attention was paid not only to statistical significance but also to practical significance in educational terms.

#### 3.3.2 Qualitative data analysis

Qualitative data were analyzed through thematic analysis, which is widely used to identify recurring patterns of meaning in participants’ accounts. The qualitative dataset consisted of semi-structured interviews conducted online with 10 primary school teachers via Zoom. All interviews were audio-recorded with participants’ consent and then transcribed verbatim. Particular care was taken during transcription to ensure that participants’ statements were transferred accurately and completely into written form, thereby minimizing data loss and supporting analytic rigor (Elo and Kyngäs, 2008).

The transcripts were imported into MaxQDA Analytics Pro 2024, which was used to organize, code, and manage the analysis process. The transcripts were read repeatedly in relation to the aims of the study and the research questions. Initial codes were generated inductively from both explicit and implicit meanings in participants’ responses. These codes were then compared, grouped, and organized into broader categories and themes. The themes were shaped in line with the semi-structured interview questions, while also allowing new meaning patterns emerging from the data to be incorporated into the analysis. This process enabled the systematic organization of the data and supported meaningful interpretations aligned with the research questions (Saldaña, 2016).

Several steps were taken to enhance the trustworthiness of the qualitative findings. First, the interview form was reviewed by three experts prior to data collection, which contributed to the content appropriateness and clarity of the questions. Second, after transcription, the interview texts were shared with participants and analysis began only after their confirmation, thereby supporting credibility through participant verification. Third, the use of a systematic coding process in MaxQDA contributed to the dependability and confirmability of the analysis by making the analytic procedure more transparent and organized. Finally, the presentation of themes together with codes, frequencies, and representative quotations in the findings section supports transferability by allowing readers to evaluate the connection between the raw data and the interpretations. During the interpretation process, attention was paid to both manifest and latent meanings, and the study’s theoretical framework and relevant literature were considered to strengthen the conceptual coherence of the findings.

The qualitative strand was designed to provide explanatory depth rather than broad generalization. Because participants were purposively selected from teachers who had prior AI training, the qualitative findings should be interpreted as information-rich accounts that help explain the quantitative results rather than as representative of all primary school teachers. For this reason, claims of broad thematic saturation should be made cautiously; the emphasis in this study is on explanatory relevance and analytic depth within an explanatory sequential mixed-methods design.

## 4 Findings

### 4.1 Quantitative findings

#### 4.1.1 Findings on primary school teachers’ perceptions of artificial intelligence in primary school mathematics instruction

##### 4.1.1.1 School mathematics instruction

Descriptive statistics for the Scale for Assessing Teachers’ Perceptions of Artificial Intelligence in Education, used in this study, are presented in Table 3. The descriptive metrics include the arithmetic mean, standard deviation, minimum, and maximum values.

**TABLE 3:** Descriptive statistics for teachers’ perception scores on the scale for assessing teachers’ perceptions of artificial intelligence in education.

Variable	Mean	SD	Min.	Max.
General	3.62	0.61	1.30	5.00
Willingness to use AI	3.98	0.80	1.00	5.00
Attitude toward AI	3.82	0.74	1.00	5.00
Professional expectations toward AI	3.66	0.60	1.40	5.00
Personal experiences with AI	3.00	0.67	1.20	5.00

The descriptive findings indicate that teachers reported moderately positive overall perceptions of AI in primary mathematics instruction (*M* = 3.62, SD = 0.61). Among the sub-dimensions, willingness to use AI (*M* = 3.98) and attitudes toward AI (*M* = 3.82) were relatively higher, whereas personal experiences with AI (*M* = 3.00) was comparatively lower. This pattern suggests that while teachers were generally open to AI and tended to view it positively at the attitudinal level, their reported practical experience with AI was more limited. Accordingly, the results appear to reflect a more favorable orientation toward AI than a deeply established pattern of classroom use.

#### 4.1.2 Comparison of scores obtained from the scale for assessing teachers’ perceptions of artificial intelligence in education by gender

The analyses conducted to compare the scores obtained from the Scale for Assessing Teachers’ Perceptions of Artificial Intelligence in Education by gender are presented in Table 4.

**TABLE 4:** Independent samples *t*-test results for teachers’ AI perception scores by gender.

Variable	Category	Mean	SD	*t*	*p*
General	Male	3.73	0.64	2.116	0.035
	Female	3.57	0.59		
Willingness to use AI	Male	4.06	0.81	1.065	0.288
	Female	3.95	0.80		
Attitude toward AI	Male	4.00	0.74	2.795	0.006
	Female	3.74	0.73		
Professional expectations toward AI	Male	3.74	0.64	1.393	0.165
	Female	3.63	0.58		
Personal experiences with AI	Male	3.13	0.79	1.868	0.064
	Female	2.95	0.62		

In the study, independent samples *t*-test results were used to examine whether scale scores differed by gender, and statistically significant differences were identified in certain dimensions. The overall mean score was calculated as 3.73 (SD = 0.64) for males and 3.57 (SD = 0.59) for females, and this difference was found to be statistically significant (*t* = 2.116; *p* = 0.035). Although the mean score for males (*M* = 4.06, SD = 0.81) was higher than that of females (*M* = 3.95, SD = 0.80) in the Willingness to Use AI dimension, this difference was not statistically significant (*t* = 1.065; *p* = 0.288). In the Attitudes Toward AI dimension, a significant difference in favor of males (*M* = 4.00, SD = 0.74) compared to females (*M* = 3.74, SD = 0.73) was observed (*t* = 2.795; *p* = 0.006). In contrast, no statistically significant gender differences were found in the Professional Expectations toward AI (Males: *M* = 3.74, SD = 0.64/Females: *M* = 3.63, SD = 0.58; *t* = 1.393; *p* = 0.165) and Personal Experiences with AI (Males: *M* = 3.13, SD = 0.79/Females: *M* = 2.95, SD = 0.62; *t* = 1.868; *p* = 0.064) dimensions. These findings indicate that gender-based differences were observed in the overall scale score and in the Attitudes toward AI dimension, with male participants scoring higher than female participants. However, such differences were not consistently found across all sub-dimensions. Therefore, the findings suggest that gender-related differences were limited to specific aspects of AI perception rather than being consistently observed across all dimensions.

#### 4.1.3 Comparison of scores obtained from the scale for assessing teachers’ perceptions of artificial intelligence in education by professional experience

The analyses conducted to compare the scores obtained from the Scale for Assessing Teachers’ Perceptions of Artificial Intelligence in Education based on professional experience are presented in Table 5.

**TABLE 5:** One-way ANOVA results for teachers’ AI perception scores by professional experience.

Variable	Category	Mean	SD	*F*	*p*	Difference
General	1–5 years	3.69	0.59	3.045	0.018	2 > 5
	6–10 years	3.81	0.58			
	11–15 years	3.62	0.62			
	16–20 years	3.66	0.70			
	21 years and more	3.43	0.50			
Willingness to use AI	1–5 years	4.11	0.71	2.749	0.029	2 > 5
	6–10 years	4.22	0.74			
	11–15 years	4.00	0.79			
	16–20 years	3.96	0.92			
	21 years and more	3.76	0.77			
Attitude toward AI	1–5 years	3.87	0.70	2.418	0.049	2 > 5
	6–10 years	4.04	0.71			
	11–15 years	3.86	0.78			
	16–20 years	3.86	0.86			
	21 years and more	3.62	0.60			
Professional expectations toward AI	1–5 years	3.78	0.59	3.438	0.009	1,2 > 5
	6–10 years	3.86	0.42			
	11–15 years	3.65	0.58			
	16–20 years	3.68	0.67			
	21 years and more	3.48	0.59			
Personal experiences with AI	1–5 years	3.00	0.72	1.585	0.178	–
	6–10 years	3.12	0.80			
	11–15 years	2.99	0.68			
	16–20 years	3.13	0.70			
	21 years and more	2.87	0.52			

1 = 1–5 years; 2 = 6–10 years; 3 = 11–15 years; 4 = 16–20 years; 5 = 21 years or more.

Professional experience was associated with statistically significant differences in the overall score and in several sub-dimensions. In general, teachers in the earlier and mid-career groups—especially those with 6–10 years of experience—reported more positive scores than teachers with 21 years or more of experience. However, this pattern was not consistent across all dimensions, as no significant difference emerged in the personal-experiences dimension. The findings therefore suggest that professional experience may be associated with certain aspects of AI perception, but not in a uniform or deterministic manner.

#### 4.1.4 Comparison of scores obtained from the scale for assessing teachers’ perceptions of artificial intelligence in education by educational level

The analyses conducted to compare the scores obtained from the Scale for Assessing Teachers’ Perceptions of Artificial Intelligence in Education by educational level are presented in Table 6.

**TABLE 6:** One-way ANOVA results for teachers’ AI perception scores by educational level.

Variable	Category	Mean	SD	*F*	*p*	Difference
General	Bachelor’s degree	3.57	0.61	2.098	0.124	–
	Master’s degree	3.68	0.61			
	Doctorate	3.83	0.56			
Willingness to use AI	Bachelor’s degree	3.96	0.84	0.815	0.444	–
	Master’s degree	3.99	0.74			
	Doctorate	4.24	0.61			
Attitude toward AI	Bachelor’s degree	3.76	0.76	1.988	0.139	–
	Master’s degree	3.90	0.70			
	Doctorate	4.05	0.73			
Professional expectations toward AI	Bachelor’s degree	3.62	0.60	1.387	0.251	–
	Master’s degree	3.72	0.61			
	Doctorate	3.84	0.45			
Personal experiences with AI	Bachelor’s degree	2.93	0.64	3.225	0.041	2 > 1
	Master’s degree	3.12	0.70			
	Doctorate	3.20	0.82			

1 = bachelor’s degree; 2 = master’s degree; 3 = doctorate.

Educational background was associated with a statistically significant difference only in the personal-experiences dimension, where teachers with a master’s degree reported higher scores than those with a bachelor’s degree. No statistically significant differences were observed in the other dimensions. Accordingly, educational background appears to be linked to only a limited aspect of teachers’ reported AI perceptions in the present study. Although doctoral-degree holders showed descriptively higher means in some dimensions, these differences were not statistically significant and should therefore be interpreted with caution, especially given the small size of that subgroup.

#### 4.1.5 Comparison of scores obtained from the scale for assessing teachers’ perceptions of artificial intelligence in education by level of AI usage

The analyses conducted to compare the scores obtained from the Scale for Assessing Teachers’ Perceptions of Artificial Intelligence in Education by the level of AI usage are presented in Table 7.

**TABLE 7:** One-way ANOVA results for teachers’ AI perception scores by frequency of AI use.

Variable	Category	Mean	SD	*F*	*p*	Difference
General	Never use	3.09	0.69	41.280	0.001	4 > 1,2,3
	Rarely use	3.49	0.49			
	Sometimes use	3.66	0.45			
	Frequently use	4.12	0.46			
Willingness to use AI	Never use	3.40	1.08	24.841	0.001	4 > 1,2,3
	Rarely use	3.85	0.70			
	Sometimes use	4.03	0.60			
	Frequently use	4.52	0.55			
Attitude toward AI	Never use	3.28	0.88	32.143	0.001	4 > 1,2,3
	Rarely use	3.65	0.61			
	Sometimes use	3.85	0.56			
	Frequently use	4.40	0.58			
Professional Expectations toward AI	Never use	3.28	0.72	17.111	0.001	4 > 1,2,3
	Rarely use	3.67	0.55			
	Sometimes use	3.64	0.49			
	Frequently use	4.01	0.51			
Personal experiences with AI	Never use	2.42	0.56	44.832	0.001	4 > 1, 2, 3
	Rarely use	2.78	0.57			
	Sometimes use	3.13	0.56			
	Frequently use	3.54	0.57			

1 = never; 2 = rarely; 3 = sometimes; 4 = frequently.

The strongest pattern in the quantitative findings was related to reported frequency of AI use. Teachers who reported using AI more frequently also tended to report more positive perception scores across all dimensions. This pattern was especially visible in the total score and in the personal- experiences dimension. At the same time, this relationship should not be interpreted causally. It is plausible that more frequent use contributes to more positive perceptions, that more positive perceptions encourage more frequent use, or that both are jointly shaped by other unmeasured factors such as digital competence, innovation orientation, or institutional support. Therefore, the results should be interpreted as indicating a strong association rather than a demonstrated directional effect.

#### 4.1.6 Comparison of scores obtained from the scale for assessing teachers’ perceptions of artificial intelligence in education by AI training status

The analyses conducted to compare the scores obtained from the Scale for Assessing Teachers’ Perceptions of Artificial Intelligence in Education based on whether participants received training on AI usage are presented in Table 8.

**TABLE 8:** Independent samples t-test results for teachers’ AI perception scores by prior AI training.

Variable	Category	Mean	SD	*t*	*p*
General	Received training	3.87	0.64	3.661	0.001
	Did not receive training	3.56	0.58		
Willingness to use AI	Received training	4.17	0.79	1.980	0.049
	Did not receive training	3.94	0.80		
Attitude toward AI	Received training	4.11	0.76	3.385	0.001
	Did not receive training	3.75	0.72		
Professional expectations toward AI	Received training	3.79	0.69	1.731	0.084
	Did not receive training	3.64	0.57		
Personal experiences with AI	Received training	3.43	0.60	5.673	0.001
	Did not receive training	2.90	0.65		

Teachers who had received prior AI-related training reported significantly higher scores than those without training in the overall score and in the willingness, attitudes, and personal-experiences dimensions. No statistically significant difference was found in the professional-expectations dimension. These findings suggest that prior training is associated with more favorable perceptions and greater self-reported familiarity with AI. However, because the data are cross-sectional and self- reported, the results should be interpreted as relational rather than causal. Training may contribute to confidence and familiarity, but it is also possible that teachers who are already more interested in AI are more likely to seek such training.

### 4.2 Qualitative findings

#### 4.2.1 Findings on the theme of perceptions toward the use of AI

Within this theme, the analysis focuses on how teachers conceptualize and evaluate AI in the context of primary school mathematics instruction. Rather than addressing specific uses or instructional procedures, this theme captures teachers’ broader interpretive positions toward AI, including the meanings, expectations, and concerns they attach to it. Participants’ views were organized under two sub-themes: Positive Perceptions and Negative Perceptions. The relevant sub-themes, codes, frequencies, and illustrative quotations are presented in Table 9.

**TABLE 9:** Themes, sub-themes, codes, frequencies, and representative quotations related to teachers’ perceptions of AI use.

Theme 1: Perceptions toward the use of AI			
Sub-theme	Code	f	Representative quotation
Positive perceptions	AI as useful and efficient	8	“I also know that in many contexts it can be life-saving… it positively affects the learning process.” (P1) “Especially from the teacher’s perspective, it greatly facilitates the process of preparing activities.” (P6)
	AI as inevitable and necessary	2	“AI is everywhere in our lives; I consider this an inevitable reality.” (P1)
	Essential for learning mathematics	1	“AI should definitely be used in mathematics, because the foundation of AI is mathematics.” (P3)
	An additional alternative for learning	1	“I see it as a third path for students. This way, they can understand better.” (P4)
	Role-dependent benefits (teacher–student)	1	“It makes planning and material preparation easier for teachers; however, it may be premature for students.” (P5) “Active use by students is more productive… It is not meaningful for teachers to use it alone.” (P9)
Negative perceptions	Infrastructure and accessibility issues	2	“The absence of a smart board at school limits applications.” (P6) “Lack of technological infrastructure can be a barrier. While some students are lucky to get access to AI easily, others don’t.” (P9)
	Accuracy–reliability concerns	2	“If the reliability of the applications has not been tested, one should be cautious.” (P2) “Beyond a certain point, the information obtained from AI becomes erroneous.” (P9)
	Age appropriateness	1	“At the primary-school level, AI remains abstract; it cannot provide tangible things to the child.” (P5)
	Insufficient teacher competencies	1	“Teachers’ lack of experience is a major problem. We are not ready yet.” (P10)

##### 4.2.1.1 Positive perceptions

Most participants view AI as a beneficial and efficient tool in education, noting that it positively influences learning processes and substantially eases teachers’ workload—particularly in activity design, lesson planning, and visual material production (P1, P6). AI is also framed as an inevitable and necessary component of contemporary education (P1). Several participants emphasized its role as an additional pathway that can address individual differences and make learning more engaging (P4). In mathematics specifically, AI was described as essential, given its mathematical underpinnings (P3). Finally, views on role-dependent benefits were mixed: some argued the advantages are more pronounced for teachers and may be premature for younger students (P5), whereas others highlighted that active student use yields greater productivity (P9).

##### 4.2.1.2 Negative perceptions

Participants also expressed reservations. At the primary-school level, AI was perceived as potentially abstract, offering limited tangible contributions to children’s learning (P5). Concerns were raised about the accuracy and reliability of AI applications, with calls for caution when tools have not been rigorously validated (P2, P9). Infrastructure and accessibility issues—such as the absence of smart boards, limited internet, or hardware constraints—were identified as practical barriers that restrict implementation (P6, P9). In addition, insufficient teacher competencies and limited AI literacy were seen as significant challenges to effective classroom integration (P10).

#### 4.2.2 Findings on the theme of stages where AI can be utilized

This theme addresses when AI is perceived to be useful within the instructional process. The analysis does not focus on the specific forms of AI use, but rather on the stages of teaching and learning in which teachers believe AI can make a contribution. Accordingly, participants’ responses were organized according to the temporal flow of instruction, including planning, lesson introduction, lesson implementation, assessment and feedback, and out-of-class learning. The related sub-themes, codes, frequencies, and illustrative quotations are presented in Table 10.

**TABLE 10:** Themes, sub-themes, codes, frequencies, and representative quotations related to the instructional stages in which AI can be used.

Theme 2: Stages where AI can be utilized			
Sub-theme	Code	f	Representative quotation
Planning process	Planning	3	“I greatly benefit from artificial intelligence during lesson preparation.” (P7) “When planning topics, I am able to adapt them according to the students.” (P5)
	Material development	1	“I particularly use it in material development by incorporating visual and auditory elements.” (P4)
Lesson introduction and opening process	Introduction activity	6	“It is something I frequently use to capture students’ attention in primary school.” (P1) “It is very effective during the attention-grabbing and motivation stages.” (P7)
Lesson implementation process	Teaching-learning process	6	“I also use artificial intelligence in the teaching and learning processes. It increases students’ interest in the lesson.” (P4) “Applications like Photomath save me time.” (P1)
	Reinforcement	3	“I use it for reinforcement activities after presenting the topic.” (P7) “I find it helpful for reinforcement in practice exercises.” (P2)
Assessment and feedback process	Assessment and feedback process	5	“Through artificial intelligence, I can identify areas where students are struggling.” (P6) “It facilitates providing feedback during assessment.” (P8)
Out-of-class learning process	Out-of-class learning	1	“It is important for students to receive support when I am not available.” (P1)

##### 4.2.2.1 Planning process

Participants most frequently emphasized using AI during pre-lesson preparation and planning. They described AI as a practical aid for structuring lessons and tailoring content to learner profiles and needs (P7, P5). In addition, AI-supported material development was noted as particularly valuable for enriching resources with visual and auditory elements that scaffold understanding and engagement (P4).

##### 4.2.2.2 Lesson introduction and opening

AI was reported to be highly effective in attention-gaining/introductory activities, especially at the primary level. Teachers stated that AI helps capture students’ interest and supports motivational set induction at the outset of lessons (P1, P7).

##### 4.2.2.3 Lesson implementation

During the teaching–learning process, participants indicated that AI tools enhance students’ interest and streamline instructional workflows (P4). Time-saving applications (e.g., problem-solving or demonstration tools such as Photomath) were frequently cited as reducing routine workload and freeing time for higher-value interactions (P1). For reinforcement, teachers use AI to provide practice opportunities and consolidate learning after initial presentation of the topic, reporting that AI-driven exercises are helpful in strengthening students’ understanding (P7, P2).

##### 4.2.2.4 Assessment and feedback

In the assessment and feedback process, participants highlighted AI’s utility in diagnosing areas where students struggle and facilitating timely, targeted feedback. AI-based analytics and response supports were viewed as improving the precision and efficiency of formative assessment (P6, P8).

##### 4.2.2.5 Out-of-class learning

Finally, participants underscored AI’s contribution to out-of-class learning, noting its value in sustaining support when the teacher is not available and enabling learners to access just-in-time assistance beyond classroom hours (P1).

#### 4.2.3 Findings on the theme of how AI is operationalized across instructional stages

While the previous theme identified the stages at which AI may be used, this theme focuses on how AI is operationalized in practice within those stages. In other words, the analysis here moves from the level of instructional timing to the level of concrete instructional enactment. Participants’ responses were grouped into sub-themes that reflect typical usage scenarios, such as content creation and presentation, problem solving and application and assessment and feedback. The relevant codes, frequencies, and sample quotations for each sub-theme are presented in Table 11.

**TABLE 11:** Themes, sub-themes, codes, frequencies and representative quotations related to how AI is operationalized across instructional stages.

Theme 3: How AI is operationalized across instructional stages			
Sub-theme	Code	f	Representative quotation
Content creation and presentation	AI for preparing questions	1	“I write the learning objective for multiplication with natural numbers and have AI generate questions.” (P10)
	Topic explanation with AI	1	“I use ChatGPT in detail for explaining topics.” (P4)
	Use of ready-made activities	1	“I support ready-made activities like GeoGebra and Kahoot with artificial intelligence.” (P4)
	Students explaining the topic using AI	1	“Students prepared the topic using ChatGPT and presented it in class.” (P3)
	Students’ making materials with AI	1	“Students can create authentic problems from daily life and generate 3D objects using AI.” (P9)
	Permanent learning through AI-generated materials	1	“It suggested visual materials to reinforce rhythmic counting, and it was effective.” (P8)
Problem solving and application	Solving questions using AI	2	“They scan the question with Photomath, and it solves step by step.” (P4) “I can guide students by using their prior knowledge.” (P10)
	Concretizing abstract concepts	1	“I use AI to concretize abstract concepts like fractions with geometric shapes.” (P2)
	Use of smart board	1	“I project it on the smart board so students can see.” (P1)
Assessment and feedback	Creating and analyzing assessment tools	1	“I have it generate tests and analyze student responses to identify gaps.” (P8)
	Personalized assessment	1	“It is possible to identify each student’s deficiencies and create personalized plans.” (P5)

##### 4.2.3.1 Content creation and presentation

Under the sub-theme of content creation and presentation, AI was reported to be used in a multifaceted manner by both teachers and students throughout the processes of content development and delivery. Participants described practices such as generating questions aligned with learning outcomes (P10), supporting topic explanations in detail through tools like ChatGPT (P4), adapting and enriching ready-made activities from platforms like GeoGebra and Kahoot with AI (P4), having students prepare content using AI and present it in class (P3), and creating 3D objects based on original, real-life problems designed by students (P9). It was also emphasized that visual material suggestions, such as those for rhythmic counting, enhance learning retention (P8).

##### 4.2.3.2 Problem solving and application

Within the sub-theme of problem solving and application, the roles of AI in accelerating the solution process and enhancing conceptual clarity stand out. It was noted that tools like Photomath enable step-by-step problem solving and allow teachers to guide students according to their prior knowledge (P4, P10). AI was reported to facilitate the concretization of abstract concepts like fractions through geometric representations (P2), which were then projected on smart boards to provide in-class visualizations (P1). These applications not only increase student engagement but also support reinforcement through practical activities.

##### 4.2.3.3 Assessment and feedback

In the sub-theme of assessment and feedback, AI is found to make concrete contributions to generating tests and similar tools, analyzing student responses to identify learning gaps (P8), and creating personalized plans for each student (P5). The findings suggest that the assessment process becomes faster, more targeted, and objective, thereby enhancing the effectiveness of formative assessment and real-time feedback.

#### 4.2.4 Findings on the theme of advantages and disadvantages of AI use for teachers and students

This theme examines what teachers believe AI changes in the educational process for both teachers and students. Unlike the previous themes, which focus on teachers’ general perceptions of AI and its place within instructional processes, this theme centers on the perceived consequences of AI use. Participants’ views were categorized into four sub-themes: advantages for teachers, disadvantages for teachers, advantages for students, and disadvantages for students. The findings are presented in Table 12 together with the relevant codes and explanatory quotations.

**TABLE 12:** Themes, sub-themes, codes, frequencies, and representative quotations related to the perceived advantages and disadvantages of AI use for teachers and students.

Theme 4: Advantages and disadvantages of AI use for teachers and students			
Sub-theme	Code	f	Representative quotation
Advantages for teachers	Reduced workload and time management	8	“It can make the teacher’s job easier and save time.” (P4)
	Professional development	3	“Artificial intelligence enhances the teacher’s creativity.” (P8)
	Facilitation of assessment and evaluation	2	“It enables us to better observe student progress.” (P6)
	Instant feedback	2	“I can more easily understand how the child has mentally coded the information.” (P3)
Disadvantages for teachers	Risk of passivity	4	“Constant use may make the teacher lazy.” (P7)
	Professional obsolescence	3	“Teachers must continuously renew themselves.” (P6)
	Accessibility challenges	3	“Lack of technological infrastructure can be a barrier. While some students are lucky to get access to AI easily, others don’t.” (P9)
	Data security concerns	1	“Data privacy is a major issue. We know that what you have searched and written on AI application is not deleted forever.” (P4)
Advantages for students	Increased motivation and attention	3	“When the teacher’s energy is high, the students also feel motivated.” (P8)
	Concretization of abstract concepts	3	“It is helpful in making abstract concepts more concrete.” (P7)
	Opportunities for individualized learning	2	“It allows students to learn at their own pace.” (P5)
	Development of higher-order thinking skills	1	“By entering commands, students develop algorithmic thinking skills.” (P6)
Disadvantages for students	Tendency toward ease and ready-made information	2	“It encourages reliance on pre-made content; students become reluctant to do research.” (P7)
	Risk of inequality in education	2	“Access to technological tools easily by some students creates injustice.” (P6)
	Deficiencies in group work and socio-emotional interaction	1	“The use of AI falls short in group-based teaching.” (P8)
	Usage difficulties among younger age groups	1	“There are challenges in use for 1st and 2nd graders. They do not know how to give prompts, which is not surprising” (P3)

##### 4.2.4.1 Advantages for teachers

Participants emphasized that artificial intelligence reduces teachers’ workload and facilitates time management; it provides automation and speed in lesson planning, activity and material development, and assessment-evaluation processes (P4, P6). As a result, more frequent and immediate feedback can be provided, and students’ cognitive representations and levels of progress can be more clearly monitored (P3, P6). Additionally, it was noted that AI supports professional development and creativity through the presentation of diverse approaches and examples (P8).

##### 4.2.4.2 Disadvantages for teachers

Participants raised concerns about the risk of passivity due to excessive or improper use of AI, highlighting the importance of teachers’ continuous self-renewal and staying up to date (P7, P6). At the school and classroom levels, hardware, connectivity, and software limitations pose accessibility challenges that hinder implementation (P9). Data security and privacy issues were also cited as significant concerns (P4).

##### 4.2.4.3 Advantages for students

It was reported that AI tools enhance student motivation and attention, and facilitate comprehension by concretizing and visualizing abstract concepts (P8, P7). The personalization features of AI, which allow students to learn at their own pace and according to their needs, were emphasized, along with its supportive role in problem-solving and higher-order thinking skills (e.g., algorithmic thinking) (P5, P6).

##### 4.2.4.4 Disadvantages for students

Participants noted that AI might increase tendencies toward ease and reliance on ready-made information, potentially reducing students’ research efforts and persistence (P7). Inequalities in access to technological tools may disrupt learning equity and pose risks of inadequacy in group work and socio-emotional interaction (P6, P8). Furthermore, difficulties in use among younger age groups (1st and 2nd grades) were highlighted (P3).

#### 4.2.5 Findings on the theme of preferences in AI-supported lesson design

This theme focuses on which AI tools and mathematical content areas teachers prioritize when designing AI-supported lessons. Rather than examining general attitudes, stages of use, or perceived effects, this theme highlights teachers’ practical preferences regarding the selection of AI-supported resources in lesson design. The findings were organized under two main sub-themes: types of AI tools to be used and subject areas in which these tools are preferred. These findings are presented in Table 13 with the corresponding codes and illustrative quotations.

**TABLE 13:** Theme, sub-themes, codes, frequencies, and representative quotations on preferences in AI-supported lesson design.

Theme 5: Preferences in AI-supported lesson design			
Sub-theme	Code	f	Representative quotation
Types of AI tools to be used	AI tools capable of solving problems	4	“I use tools like GeoGebra, AI Mathematics, Photomath, Mathigon, and ChatGPT. They are very helpful in solving problems.” (P4, P10)
	AI tools generating visuals or videos	4	“With tools like Leonardo AI, it becomes easier to visualize data and demonstrate the unfolding of geometric shapes.” (P5) “With tools like Sora, I can present mathematical topics as animations and videos to students.” (P8)
		
	AI tools with free plans	3	“Access to free tools like Gemini and Bing is easy. ChatGPT also offers a free plan, though limited.” (P6)
	AI tools that can explain math topics	3	“Tools like ChatGPT, Bing, and Gemini assist directly with topic explanations. They capture students’ attention.” (P7)
	AI tools for problem scenario generation	3	“They can generate problems from given elements in math. They provide a variety of problem scenarios.” (P4)
	Game-based AI tools	3	“Game-based applications like Mathigon and Matific attract students’ interest.” (P8)
	AI tools for assessment	2	“Applications like DragonBox can be used for evaluating student data and reinforcing learning.” (P5)
	AI tools for designing lesson stages	1	“Lesson design can be done with Bing, Gemini and ChatGPT.” (P5)
Subject areas	Four operations and problem solving	10	“AI saves time when teaching multiplication tables in Grade 3 and can guide problem solving in Grade 4.” (P6, P9)
	Geometry	1	“I would prefer to use AI to concretize geometric shapes in Grade 1.” (P9)
	Measurement (e.g., time)	1	“AI is effective in concretizing the topic of clocks.” (P2)
	Numbers and place value	1	“I use AI when teaching place value in Grade 2.” (P9)

##### 4.2.5.1 Types of AI and AI-supported digital tools to be used

Most participants found AI and AI-supported digital tools particularly beneficial in the teaching process for problem-solving and concretization functions. Tools such as GeoGebra, Photomath, Mathigon, and ChatGPT, which are capable of supporting problem solving, facilitate the teaching process by offering students step-by-step solution paths and enabling them to develop various strategies (P1, P4, P9, P10). Additionally, tools such as Leonardo AI and Sora, which generate visuals and videos, help students better understand abstract concepts, capture their attention, and increase engagement with learning (P5, P8). Participants also emphasized that the ease of access to tools with free plans, such as Gemini and Bing, boosts their motivation to use these tools (P5, P6, P8). Furthermore, tools that explain topics, generate problem scenarios, and provide game-based learning experiences stand out for making lessons more interactive and engaging (P2, P6, P9, P10).

##### 4.2.5.2 Subject areas

The majority of participants stated that AI-supported applications are particularly suitable for supporting instruction in the four basic operations and supporting problem-solving processes. AI tools present students with step-by-step operational sequences and offer alternative solutions, thereby strengthening both procedural fluency and strategic thinking in problem solving (P3). It was specifically noted that these tools save time and provide guidance in teaching multiplication tables in Grade 3 and in problem-solving activities in Grade 4 (P6, P9). This indicates that the domain of four operations and problem solving is the most frequently preferred area in AI-supported instruction.

## 5 Discussion and conclusion

The present study examined primary school teachers’ reported perceptions of artificial intelligence (AI) in mathematics instruction, differences in these perceptions according to selected background and AI-related variables, and teachers’ accounts of the pedagogical roles, opportunities, and constraints associated with AI use. Taken together, the quantitative and qualitative findings portray a cautiously favorable orientation toward AI rather than evidence of deeply established classroom integration. Teachers generally reported positive attitudes and willingness to use AI, yet their personal experience scores were comparatively lower. Their qualitative accounts further showed that AI was primarily positioned as a supportive resource for planning, material development, visualization, problem solving, assessment, feedback, and out-of-class learning. At the same time, participants repeatedly raised concerns about infrastructure, reliability, data privacy, teacher competence, age-appropriateness, and students’ possible over-reliance on ready-made solutions. This combination of perceived pedagogical value and practical caution provides an important context for interpreting the individual research questions.

### 5.1 Discussion of the first research question

The first research question concerned teachers’ reported perceptions of AI in primary mathematics instruction. The overall pattern was moderately positive, with willingness to use AI and attitudes toward AI receiving comparatively higher scores than personal experiences. Rather than indicating uniformly established adoption, this pattern suggests a distinction between being receptive to AI and having extensive experience integrating it into instructional practice. Recent K–12 research points to a similar distinction. Cheah and Kim (2026) found that STEM teachers varied considerably in their familiarity with generative AI and that greater familiarity was positively associated with more favorable perceptions of its educational integration. Similarly, Mah et al. (2026) reported that K–12 teachers were still at a relatively early stage of AI integration and that AI-related professional development opportunities had not yet been extensively used. The present findings therefore appear to reflect a context in which teachers may recognize the potential value of AI before its use becomes routine or pedagogically consolidated.

The qualitative findings help explain this apparent attitude–experience gap. Teachers described AI as useful for reducing workload, preparing materials, creating visual resources, differentiating content, supporting problem solving, and providing feedback. At the same time, they identified limitations concerning infrastructure, reliability, teacher competence, and the suitability of AI for younger learners. A similar combination of perceived affordances and implementation barriers has been documented in recent studies of practicing teachers. Filiz et al. (2025), in a study of K–12 teachers in Türkiye, found that teachers associated AI with efficiency, adaptability, personalization, interactivity, and support for lesson planning, but also identified technical, pedagogical, curricular, and ethical barriers to its integration. Cheah et al. (2025) likewise showed that K–12 teachers’ interest in generative AI coexisted with preparedness gaps and barriers to classroom implementation. Thus, positive perceptions should not be treated as equivalent to extensive or pedagogically mature use.

This interpretation is also compatible with recent work on AI acceptance and literacy. Deshen et al. (2026) found positive associations between teachers’ AI acceptance variables and AI literacy, with willingness to use AI among the strongest predictors of AI literacy in their sample. Although the present study did not directly measure AI literacy and therefore cannot infer such a relationship, the relatively high willingness scores together with comparatively lower personal-experience scores suggest that acceptance-related orientations and practical experience may develop at different rates. From the conceptual perspective adopted in the present study, TAM is useful for interpreting teachers’ favorable orientations toward adoption, whereas TPACK-related perspectives draw attention to the additional pedagogical, technological, and content-specific knowledge required to translate such orientations into classroom practice. This distinction is particularly important in mathematics, where AI-generated explanations or solutions must be evaluated not only for technical accuracy but also for their alignment with students’ conceptual development and instructional goals.

Evidence specifically from primary mathematics education reinforces this interpretation. Li and Manzari (2025), working with 516 primary mathematics teachers, found that both internal factors—including teachers’ attitudes and TPACK—and external factors such as institutional resources and support were associated with AI utilization. Although their context and design differ from those of the present study, their findings support the broader view that favorable attitudes constitute only one component of AI integration. The current results similarly suggest that teachers’ openness to AI exists alongside contextual and competency-related conditions that may shape whether and how that openness is translated into reported practice.

### 5.2 Discussion of the second research question

The second research question examined differences in teachers’ AI perceptions according to gender, professional experience, educational background, frequency of AI use, and prior AI training. An important feature of the findings is that these variables did not produce a uniform pattern. Gender, professional experience, and educational background were associated with particular dimensions, whereas frequency of AI use and prior AI training showed the strongest and most consistent relationships with teachers’ perception scores. This distinction is important because it suggests that variables reflecting teachers’ direct engagement with AI may be more closely associated with their perceptions than demographic characteristics alone.

Gender differences were statistically significant for the overall perception score and the attitudes dimension, with male teachers reporting higher scores, but no significant differences emerged for willingness, professional expectations, or personal experiences. The limited nature of this pattern warrants caution. Broader research on AI adoption has reported gender differences in AI attitudes and anxiety; for example, Russo et al. (2025) found that women reported greater AI anxiety and less positive AI attitudes than men. However, teacher-specific evidence is not consistent. In Türkiye, Çayak (2024), in a study of 361 teachers, found no significant gender differences in positive or negative AI attitudes or AI literacy. Accordingly, the present gender result is better interpreted as a dimension-specific association within this sample than as evidence of a general gender effect on teachers’ AI perceptions. Factors not directly measured in this study, including prior exposure, confidence, AI anxiety, or patterns of technology use, may contribute to such differences and should be examined directly before stronger explanations are advanced.

Professional experience showed a similarly differentiated pattern. Teachers with 6–10 years of experience tended to report more favorable perceptions than those with 21 or more years of experience in several dimensions, but the pattern was not consistent across the scale and no significant difference emerged for personal experiences. Moreover, Çayak (2024) found no significant differences in teachers’ AI attitudes or literacy according to professional seniority. These inconsistencies caution against explaining AI perceptions primarily in terms of years of teaching experience. Professional experience may overlap with other factors, such as opportunities to participate in technology-related professional development, institutional context, frequency of digital-tool use, and willingness to experiment with emerging technologies. Since these factors were not simultaneously modeled in the present study, professional experience should be regarded as an associated background characteristic rather than a direct explanation for AI acceptance.

Educational background was associated only with the personal-experiences dimension, where teachers with a master’s degree reported higher scores than those with a bachelor’s degree. The absence of significant differences across the other dimensions suggests that postgraduate education was not associated with a broadly more favorable perception of AI in this sample. Rather, it may reflect differences in reported exposure or experience. This interpretation is consistent with the broader pattern of the study: direct or indirect opportunities to engage with AI appear more relevant than demographic or credential-based distinctions alone. Nevertheless, because the doctoral subgroup was small and the study did not examine the content of participants’ postgraduate education, the educational-background finding should not be interpreted as evidence that additional academic qualifications themselves lead to stronger AI engagement.

The clearest quantitative pattern concerned frequency of AI use. Teachers who reported using AI more frequently also reported more positive perceptions across all dimensions, with particularly marked differences in overall scores and personal experiences. This result is consistent with Cheah and Kim’s (2026) finding of a positive relationship between STEM teachers’ familiarity with GenAI and their perceptions of its integration in education. Russo et al. (2025) also reported an association between prior AI use and more positive AI attitudes, although their sample was not limited to teachers. Taken together, these findings suggest that experience with AI and positive perceptions frequently co-occur. However, the direction of this relationship cannot be established from the present cross-sectional data. More frequent use may increase familiarity and reduce uncertainty, but teachers who already view AI positively may also be more inclined to use it. Both patterns may additionally be influenced by factors such as AI literacy, digital competence, institutional support, or access to appropriate tools.

Prior AI training showed a similarly strong association. Teachers who had received AI-related training reported higher overall scores and higher willingness, attitudes, and personal-experience scores than teachers without such training, although the professional-expectations dimension did not differ significantly. This result is compatible with emerging evidence emphasizing professional learning as an important correlate of teacher engagement with AI. Runge et al. (2025), working with preservice teachers, found that AI-related teacher training and AI-TPACK were relevant to understanding AI acceptance. More recently, Mah et al. (2026) reported that participation in and satisfaction with AI-related professional development among K–12 teachers were positively associated with several dimensions of perceived instructional quality. These studies differ in population and outcome variables, but together they reinforce the relevance of structured opportunities to learn about AI.

The qualitative findings provide additional context for this pattern because participants explicitly identified limited teacher competence and AI literacy as barriers to classroom integration. Thus, the quantitative association between prior training and more positive perceptions corresponds with teachers’ qualitative emphasis on competence as a condition shaping their engagement with AI. Nevertheless, the present data do not establish that training caused the observed differences. Teachers who are already interested in AI may be more likely to participate in available training, and the study did not examine the duration, content, or quality of the training participants had received. The most defensible conclusion is therefore that prior training and more frequent AI use were the variables most consistently associated with favorable AI perceptions in this sample, rather than that either variable independently produces positive attitudes.

### 5.3 Discussion of the third and fourth research questions

The third and fourth research questions addressed how teachers positioned AI within primary mathematics instruction and the opportunities and constraints they associated with its use. A central qualitative finding was that teachers did not describe AI as serving a single instructional function. Instead, they reported using or envisioned using AI across multiple stages of teaching, including planning, lesson introduction, implementation, reinforcement, assessment and feedback, and out-of-class learning. Planning was particularly prominent, with teachers referring to lesson preparation, differentiation, question generation, and the production of visual and auditory materials. This teacher-facing orientation corresponds closely with recent K–12 evidence. Filiz et al. (2025) found lesson planning and the creation or adaptation of instructional materials among the functions teachers associated with AI, while Cheah et al. (2025) documented a range of teacher practices involving generative AI alongside concerns about preparedness for classroom integration.

In mathematics specifically, the findings point to a form of AI use in which teachers remain responsible for selecting, interpreting, and adapting technological output. Participants described generating questions, developing explanations, visualizing mathematical ideas, adapting digital activities, and using AI-supported tools during problem solving. This is consistent with recent mathematics-education research emphasizing teacher–AI interaction rather than simple automation. Segal and Klemer (2025), for example, examined mathematics teachers’ dialogic interactions with GenAI during task design and considered these interactions in relation to TPACK. Galiç et al. (2025), in scenario-based training with in-service mathematics teachers, used ChatGPT as a virtual student to examine teacher noticing of mathematical rationality. Their findings illustrate that generative AI can become an object of professional interpretation and pedagogical reasoning rather than merely a source of ready-made content. In this respect, the present participants’ descriptions of adapting AI-supported materials and guiding students through generated solutions are consistent with a teacher-mediated conception of AI use.

Problem solving and the concretization of mathematical ideas were especially prominent in participants’ accounts. Tools such as ChatGPT, Photomath, GeoGebra, and Mathigon were associated with step-by-step solutions, alternative strategies, visualization, and support for abstract concepts such as fractions. Participants particularly favored AI and AI-supported digital tools for the four basic operations and problem-solving activities. This preference is understandable in relation to the affordances of digital mathematics tools, but the findings should be interpreted as teachers’ perceptions of usefulness rather than evidence that these tools improved students’ mathematical learning. Hwang’s (2022) meta-analysis found a positive but relatively small overall effect of AI-supported interventions on elementary students’ mathematics achievement, indicating that AI can contribute to learning under some conditions but should not be understood as uniformly or independently effective. More recent synthesis focusing specifically on ChatGPT in school mathematics similarly identifies both educational opportunities and substantial pedagogical challenges (Turmuzi et al., 2026). The present study therefore adds evidence about where practicing teachers perceive AI to be useful, rather than testing whether those perceived functions lead to improved achievement.

Assessment and feedback formed another important area of perceived use. Teachers described AI as supporting question generation, identifying learning gaps, providing rapid feedback, and helping develop personalized learning plans. These accounts are consistent with mathematics-education research on data-supported formative assessment. Moed-Abu Raya and Olsher (2024) demonstrated how learning analytics visualizations can become part of mathematics teachers’ formative assessment practices and instructional decision-making. In the present study, however, the evidence concerns teachers’ reported and anticipated uses rather than observed assessment practices. Consequently, claims that AI makes assessment more objective or effective should be understood as participant perceptions. What the findings more directly demonstrate is that teachers see assessment and feedback as one of the instructional areas in which AI may provide practical support.

The perceived advantages for teachers were closely related to efficiency. Participants emphasized reduced workload, time savings, faster preparation of materials, and more immediate feedback. At the same time, they did not describe efficiency as unconditionally positive. Concerns were expressed that excessive reliance on AI could contribute to teacher passivity and weaken professional creativity. This tension is important because it differentiates the use of AI as professional support from the delegation of pedagogical judgment. Recent K–12 research similarly suggests that the challenge is not simply whether teachers use AI but whether they possess the knowledge needed to critically evaluate and adapt its outputs (Filiz et al., 2025; Mah et al., 2026). From a TPACK perspective, the pedagogical value of AI therefore depends on the interaction between technological affordances, mathematical content, and instructional judgment rather than on automation itself.

Participants identified a comparable tension for students. On the positive side, they associated AI with motivation, attention, personalization, visualization, problem-solving support, and opportunities to learn at an individual pace. On the negative side, they were concerned that students might become dependent on ready-made information, invest less effort in research or problem solving, and have fewer opportunities for independent reasoning. These concerns are especially significant in mathematics because access to a correct or fluent solution does not necessarily entail understanding the reasoning that produces it. Turmuzi et al. (2026) similarly identify concerns about inaccurate outputs, overdependence, and the need for careful pedagogical orchestration in school mathematics uses of ChatGPT. The present findings extend this issue to primary education by showing that teachers are particularly sensitive to the possibility that easy access to solutions may conflict with the development of persistence and independent mathematical thinking.

Age-appropriateness emerged as a further boundary condition. Some teachers questioned the direct usefulness of AI for younger primary students and specifically identified difficulties for first- and second-grade learners. This finding should not be generalized into a claim that AI is unsuitable for younger children, since the study did not compare AI use across grade levels or observe students interacting with these technologies. Rather, it shows that developmental appropriateness is part of teachers’ decision-making about AI use. Such caution is consistent with the broader teacher-mediated pattern identified throughout the qualitative findings: participants often appeared more comfortable with AI as a resource used by the teacher to design, visualize, adapt, or assess instruction than with unrestricted direct use by younger students.

Finally, infrastructure, accessibility, reliability, privacy, and teacher competence were repeatedly identified as constraints. These concerns indicate that teachers’ perceptions of AI integration are embedded within the material and institutional conditions of schooling. The finding is particularly consistent with Li and Manzari’s (2025) study of primary mathematics teachers, in which AI utilization was associated not only with internal factors such as attitudes and TPACK but also with external conditions including institutional resources and support. Filiz et al. (2025) similarly identified technical and contextual barriers among K–12 teachers in Türkiye. In the present study, these constraints help explain why positive attitudes and willingness to use AI can coexist with more limited personal experience. They also reinforce the need to distinguish teachers’ recognition of potential affordances from evidence of sustained classroom implementation.

### 5.4 Conclusion

The findings of this study indicate that primary school teachers in the sample generally viewed AI in mathematics instruction favorably but did not report an equally developed level of personal experience. This difference between relatively high willingness and attitudes and lower personal-experience scores was reinforced by the qualitative findings, in which participants described multiple potential and reported uses of AI while simultaneously identifying practical, pedagogical, and ethical constraints. Among the background variables examined, gender, professional experience, and educational background were associated with particular dimensions rather than producing consistent differences across AI perceptions. In contrast, frequency of AI use and prior AI training showed the clearest and most consistent associations with more favorable perception scores; because the study was cross-sectional, these relationships should not be interpreted causally.

Teachers primarily positioned AI as a supportive resource across lesson planning, material and content development, visualization, problem solving, reinforcement, assessment, feedback, and out-of-class learning. AI and AI-supported tools were particularly associated with the four basic operations and problem solving, while participants also emphasized potential benefits related to efficiency, differentiation, visualization, and timely feedback. At the same time, they identified concerns regarding infrastructure, access, reliability, privacy, teacher competence, age-appropriateness, and student reliance on ready-made solutions. Accordingly, the contribution of the present study is not to demonstrate that AI improves mathematics teaching or student learning, but to show how a group of primary school teachers in Türkiye currently perceive and describe its place within mathematics instruction. Their accounts portray AI primarily as a teacher-mediated instructional resource whose perceived usefulness coexists with clear reservations about how, when, and for whom it should be used. Further evidence based on observed classroom practice and student outcomes is needed before stronger conclusions can be drawn about the quality or effectiveness of AI integration in primary mathematics education.

## 6 Limitations

Several limitations should be considered when interpreting the findings of this study. First, the quantitative sample consisted of 302 primary school teachers recruited through voluntary online participation and convenience sampling. Although the participants represented different levels of professional experience, educational backgrounds, and AI-use profiles, the sample was not designed to be statistically representative of primary school teachers in Türkiye. Teachers with greater interest in digital technologies or AI may also have been more likely to participate, creating the possibility of self-selection bias.

The second point is that both strands of the study relied primarily on self-reported data. Survey responses captured teachers’ reported perceptions, while the interviews reflected participants’ descriptions and interpretations of AI-supported mathematics teaching. Such data may be influenced by recall limitations, social desirability, or discrepancies between reported and actual classroom practice. The study therefore provides evidence about how teachers perceive and describe AI use rather than direct evidence of the frequency, quality, or depth of AI integration in classroom settings.

Thirdly, the cross-sectional design limits causal and developmental interpretation. Associations between AI-use frequency, prior AI training, and more favorable perceptions cannot establish the direction of influence. Teachers who use AI more frequently or participate in AI-related training may develop greater familiarity and more positive perceptions, but teachers who already hold favorable attitudes may also be more likely to use AI and seek relevant training. In addition, potentially relevant factors such as AI literacy, digital competence, innovation orientation, school-level support, and access to technology were not directly measured. Longitudinal research would be needed to examine how teachers’ perceptions and practices develop as their experience with AI increases.

Another limitation is that the qualitative phase involved 10 teachers purposively selected from participants who had previously received AI-related training. This strategy was appropriate for obtaining information-rich accounts capable of elaborating the quantitative findings, but it also means that the qualitative strand primarily reflects the perspectives of teachers with some prior exposure to AI. Teachers without AI-related training may perceive different opportunities, barriers, or professional learning needs. Accordingly, the qualitative findings should be understood as explanatory and contextually situated rather than representative of the wider population of primary school teachers.

Although the scale demonstrated very high internal consistency in the present sample, the total-scale Cronbach’s alpha coefficient was 0.954. While this supports score reliability, coefficients of this magnitude may also indicate substantial overlap among items. Additional construct-level analyses, including subscale reliability estimates, corrected item–total correlations, and factor-analytic evidence based on independent samples, would provide a more comprehensive evaluation of the measurement properties of the instrument.

Finally, the study did not directly examine student achievement, mathematical reasoning, classroom interaction, instructional quality, or observed outcomes associated with AI-supported teaching. Consequently, teachers’ reports that AI may support visualization, problem solving, differentiation, feedback, or student engagement should not be interpreted as evidence that these outcomes actually occurred. The findings instead identify the pedagogical functions and concerns that teachers associate with AI in primary mathematics instruction.

## 7 Implications and recommendations

The findings have implications for classroom practice and teacher professional learning, but these implications should be interpreted within the perception-based and self-reported nature of the study. Teachers most frequently positioned AI as a supportive resource for lesson planning, material development, visualization, problem solving, reinforcement, and formative assessment rather than as a substitute for teacher judgment. This suggests that classroom applications of AI may be most appropriately considered in relation to specific instructional purposes. In primary mathematics, teachers may use AI-supported tools to generate differentiated practice, develop alternative representations of mathematical ideas, prepare questions and materials, or assist with feedback, while retaining responsibility for evaluating the mathematical accuracy, developmental appropriateness, and pedagogical value of generated outputs.

Participants’ concerns about students’ reliance on ready-made responses also indicate the importance of maintaining opportunities for mathematical reasoning and independent problem solving. In this respect, AI-supported activities should not be designed solely around obtaining faster solutions. Particularly when tools provide step-by-step answers, teachers may need to structure tasks so that students are required to explain reasoning, compare strategies, justify solutions, identify errors, or revise AI-generated responses. Such uses would be more consistent with the participants’ view of AI as a supportive rather than substitutive resource and with their concerns about passivity and reduced persistence.

The association between prior AI training and more favorable perceptions, together with participants’ concerns about limited AI competence, points to the potential value of professional learning opportunities that extend beyond basic tool familiarization. Professional development may be more useful when it addresses how AI-supported tools can be aligned with mathematical learning objectives, how generated content and solutions can be critically evaluated, and how teachers can make age-appropriate decisions about direct student use. Issues identified by participants—including reliability, privacy, accessibility, and over-reliance—also suggest that ethical and critical evaluation should form part of such professional learning rather than being treated separately from pedagogical use. Because the present study did not evaluate the effects of professional development, however, these implications should be understood as priorities suggested by the findings rather than as evidence of the effectiveness of any particular training model.

The reported infrastructure and accessibility barriers similarly indicate that teachers’ willingness to use AI may not, by itself, be sufficient for classroom implementation. Participants referred to internet connectivity, hardware availability, software access, and unequal technological opportunities as practical constraints. School- and system-level planning for AI-supported instruction should therefore take account of the material conditions under which teachers and students are expected to use these technologies. The present findings do not establish which infrastructural investments are most effective, but they show that teachers perceive access and reliability as relevant conditions shaping their ability to engage with AI-supported practices.

The findings also suggest several directions for future research. Studies employing classroom observations, teacher activity logs, instructional artifacts, or screen-recorded interactions with AI tools could examine whether and how teachers’ reported practices correspond to actual classroom enactment. Research involving students could investigate how different forms of AI-supported instruction relate to mathematical reasoning, problem-solving processes, conceptual understanding, motivation, and achievement. Such studies would be particularly valuable in primary education, where developmental appropriateness and the balance between technological support and independent thinking require closer empirical examination.

Longitudinal and intervention-based research is also needed to clarify the relationships observed in the present study. In particular, following teachers before and after sustained AI-related professional learning could help determine whether changes in familiarity, AI-related knowledge, and classroom experience are accompanied by changes in perceptions and instructional practice. Future studies could additionally measure variables such as AI literacy, AI-TPACK, digital competence, AI anxiety, institutional support, and school-level technological capacity in order to examine how these factors interact with AI use and teacher perceptions. Including teachers with and without prior AI training in the qualitative phase would further broaden understanding of the range of perspectives represented in primary education.

Overall, the practical implication of the study is not that AI should be adopted more extensively simply because teachers generally view it positively. Rather, the findings suggest that teachers distinguish between useful instructional support and potentially problematic use. Their accounts emphasize purposeful use, teacher mediation, age-appropriate implementation, critical evaluation of AI outputs, and attention to the practical conditions of access. Future practice and research can build on these concerns by examining not only whether AI is used in primary mathematics classrooms, but how it is integrated and what forms of use support meaningful mathematical learning.

## Data Availability

The original contributions presented in the study are included in the article/supplementary material, further inquiries can be directed to the corresponding authors.
